# Defeat of the Anatomy-Bill, with Reflexions

**Published:** 1829-07-01

**Authors:** 


### Mr. Warburton's Bill.

The bill for legalising dissection of human bodies, and for facilitating the study of anatomy, has been withdrawn, or, in other words, lost, in the House of Lords. It requires not the gift of prophecy to foretel that the whole measure is lost with it—for, if any other bill be introduced and passed during the ensuing session, (which we very much doubt,) it will be saddled with such restrictions, and crippled with such difficulties as will nullify any salutary operation that may be expected from it. The late abortive attempt in Parliament has rendered more manifest to the public than ever, the wretched and distracted state of the medical profession in this country! Such an anomalous and mis-shapen monster has never been fabled by poet's imagination—

"Monstrum horrendum informe, ingens cui lumen ademptum."

If we were called upon for a definition or brief character of it, we would say that it consists of—"three BODIES without a HEAD—and some thousands of MEMBERS unattached to any of the BODIES—except by some shreds of *parched* skin." That such a heterogeneous monster should be capable of any combined, simultaneous, or regularly progressive movement, is not to be expected. The late transaction has afforded a fine illustration. The anomalous animal in question did feel aggrieved, and contrived, though with much difficulty and aukwardness, to make its grievances known—at first through its MEMBERS, and ultimately through its BODIES. There was more unity of sentiment respecting the nature, cause, and remedy of the malady, than might have been expected—but no sooner was a prescription offered for the disease, comprehending the main remedial agents for its removal, than a hue and cry was set up about the formula of the prescription, and about certain trifling and unimportant ingredients in the mixture, which completely puzzled the doctor, and caused all thoughts of cure to be abandoned!

To drop metaphor. The profession has long laboured under the difficulties attendant on the study of Anatomy, and at length induced the legislature to entertain a measure for their relief. A zealous member of the House of Commons, and a liberal committee agreed on the *fundamental principles* of a bill for the measure in question—these fundamental principles being the legalising of dissection, and of surrendering unclaimed bodies for the purposes of anatomy. These principles formed the prayer of every petition to the Legislature from the members and the bodies corporate of the profession. Well! A bill was prepared embodying and embracing these principles, but encumbered with certain regulations which were of little importance, compared, at least, with the good which was to be obtained. The bill passed the House of Commons; but before it could reach the House of Lords, the members of the profession were all in arms about the minor enactments of the bill—and the POPULAR PRESS was actually fed, from day to day, by traitors from the ranks of medical society, with the most inflammatory attacks on the fundamental principles of the bill! The business was not confined to covert acts. The medical press added fuel to the flame—and, strange as it may seem, petitions against the bill were, at one and the same moment, on the table of the House of Lords, from Churchwarden Wakley and the College of Surgeons! The ground of Mister Wakley's opposition was, that the bill would give increased power to the College of Surgeons—the opposition of the College was founded on the supposition that the bill would deprive that body of all its best privileges and powers! Such a monstrous coalition as that of WAKLEY and the COLLEGE of SURGEONS, must end either in an abortion, or in the evolution of some shapeless, inhuman, and caliban birth, such as a Shakespeare only could shadow forth. We do not deem it necessary to expend much time or space on this matter. We may only observe that the want of unity—in other words, the want of a HEAD or of a HEART in the medical profession, by which any measure of amelioration could be properly and quietly urged on the attention of the Legislature has given rise to such a profusion of *popular* appeals to the public, as to raise a host of prejudices among the *vulgar*, in all ranks of society, that will not be easily subdued.

We hazard then a prophecy—that so good a bill as that which has been rejected will never again pass the Legislature—at least in our days! And, as a comfort to Churchwarden Wakley, we prophecy that, *if* a bill come forward in the next session of Parliament, it will be under the auspices of the COLLEGE of SURGEONS—and will give to THAT BODY ten times the powers and privileges which he, in his usual ignorance and perversity of intellect, supposed the late bill would have given! Let him register this prophecy, and upbraid us with its non-fulfilment, if he can.

